# Visuo-spatial attention deficit in children with reading difficulties

**DOI:** 10.1038/s41598-022-16646-w

**Published:** 2022-08-17

**Authors:** Sandro Franceschini, Sara Bertoni, Giovanna Puccio, Simone Gori, Cristiano Termine, Andrea Facoetti

**Affiliations:** 1 Developmental and Cognitive Neuroscience Lab, Department of General Psychology, University of Padua, Via Venezia 8, 35131 Padua, Italy; 2 Child Neuropsychiatry Unit, Department of Medicine and Surgery, University of Insubria, Varese, Italy; 3 Department of Human and Social Sciences, University of Bergamo, Bergamo, Italy

**Keywords:** Psychology, Human behaviour, Attention, Dyslexia, Perception, Reading

## Abstract

Although developmental reading disorders (developmental dyslexia) have been mainly associated with auditory-phonological deficits, recent longitudinal and training studies have shown a possible causal role of visuo-attentional skills in reading acquisition. Indeed, visuo-attentional mechanisms could be involved in the orthographic processing of the letter string and the graphemic parsing that precede the grapheme-to-phoneme mapping. Here, we used a simple paper-and-pencil task composed of three labyrinths to measure visuo-spatial attention in a large sample of primary school children (n = 398). In comparison to visual search tasks requiring visual working memory, our labyrinth task mainly measures distributed and focused visuo-spatial attention, also controlling for sensorimotor learning. Compared to typical readers (n = 340), children with reading difficulties (n = 58) showed clear visuo-spatial attention impairments that appear not linked to motor coordination and procedural learning skills implicated in this paper and pencil task. Since visual attention is dysfunctional in about 40% of the children with reading difficulties, an efficient reading remediation program should integrate both auditory-phonological and visuo-attentional interventions.

## Introduction

Developmental dyslexia is a specific reading disorder despite normal intelligence, teaching experience and absence of any manifest sensory deficit. The diagnostic and statistical manual for mental disorders (DSM-5) classifies developmental dyslexia as a possible outcome of a specific learning disorder. The diagnosis of a specific learning disorder is often accompanied or preceded by other diagnoses within the group of neurodevelopmental disorders, such as language disorder, attentional deficit and hyperactivity disorder, developmental coordination disorder and autism spectrum disorder^1^.

The phonological core deficit theory argues that reading difficulties (RD) in children with developmental dyslexia stems from deficits in the ability to identify and explicitly act upon sounds of spoken words, leading to difficulties in learning appropriate grapheme to phoneme mapping^2,3^. Thus, the left temporoparietal junction, involved in auditory-phonological processing and memory of the speech sounds^4^, could play a crucial role in the first phases of reading acquisition in which grapheme-to-phoneme mapping plays a pivotal role in the sub-lexical and lexical route development^5^.

The time required for a prereading child to quickly and accurately name an array of well-known visual stimuli, known as rapid automatized naming (RAN), is one of the best predictors of future reading skills^6,7^. Exactly as reading, RAN tasks require: (i) attention to the stimuli; (ii) visual processes that are responsible for initial feature detection, discrimination, and stimuli identification; (iii) integration of visual information with stored orthographic and phonological representations; (iv) lexical processes, including access and retrieval of phonological codes; and (v) organization of articulatory output^8^. Longitudinal studies have shown that phonological awareness and visuo-spatial working memory, as well as RAN, appear to be good predictors of future reading development^2,7,9,10^.

The fronto-parietal network, involved in visuo-attentional processing, could play a crucial role in the first phases of letters identification and orthographic development^11,12^. Efficient abilities in extraction and selection of the visual information through visuo-spatial attention allow to create stronger visual word form representations^13^ (see^14–17^ for reviews; see^18^ for a meta-analysis). As reported by Grainger et al. (p. 171)^17^: “processing of orthographic information begins with scale-invariant gaze-centered letter detectors that conjunctively encode letter identity and letter location. Visual acuity, crowding, and spatial attention conjointly determine activity in these gaze-centered letter detectors”.

Combining signal-enhancing and noise-exclusion mechanisms, the right fronto-parietal network is involved in attentional shifting (i.e., disengagement of attentional focus) and scaling (zoom-in and zoom-out of attentional focus)^19^.

The presence of sluggish attentional shifting^20–24^, as well as an altered perceptual noise-exclusion mechanism^25,26^, could be at the basis of difficulties in rapid stimulus-sequence processing often observed in children with developmental dyslexia^14^. Several longitudinal studies have confirmed that visuo-spatial attention abilities are good predictors of future reading skills^26–29^ (see^18^ for a meta-analysis in pre-reading children), suggesting that graphemic parsing and letter-string processing demand: (i) rapid and accurate deployment of visual attention along the letter strings^17,30,31^; (ii) good abilities in global extraction and spatio-temporal integration of visual information^32–34^; (iii) a large visual-attention span and; (iv) a reduced visual crowding effect^26,30^ (see^35^ for a review). Crucially, visuo-attentional training appears to improve reading skills in children with and without developmental dyslexia^26,33,34,36–42^ (see^43^ for a review), confirming the causal role of the fronto-parietal attentional network in reading acquisition^44^.

The fronto-parietal network is involved not only in attentional deployment crucial for reading acquisition but also in multiple interactions with the environment, particularly in action recognition and action planning^45^. It is intriguing to note that children with developmental dyslexia show a rate of comorbidity from 16 to 70% with a developmental coordination disorder^46,47^ and that this neurodevelopmental disorder is characterized by visuo-attentional deficits^48^. However, despite the evidence about the correlation between visuo-motor and reading and spelling skills achievement^49,50^, some longitudinal studies on the possible links between fine and gross motor skills and reading development have not shown consistent results^2,29^. Specifically, some studies linked manual dexterity to future reading skills when evaluated with writing tasks^51^. However, this correlation is sometimes better explained by attentional and working memory functions^49,52^, and sometimes manual dexterity does not appear to be related to future reading skills^53^. A possible explanation for these inconsistent results could be the different levels of engagement of visuo-attentional mechanisms involved in the specific experimental tasks^54^.

In children with developmental dyslexia, a sequential procedural learning (the ability to acquire a general task procedure) deficit has also been observed mainly in serial reaction time tasks^55^. Interestingly, in their meta-analysis, Lum and colleagues^56^ showed that the observed deficit appears to be mainly linked to a possible dysfunction in medial-temporal areas, involved in the attentional spatiotemporal sequence processing. Also, in tasks that strongly involve visuo-hand coordination, such as mirror drawing, children with developmental dyslexia show slower execution times^55,57^. Although visuo-motor coordination deficits and procedural learning disorders have been shown in developmental dyslexia, a basic visuo-attentional deficit could indirectly explain these motor and learning disorders^14^.

**Figure 1:**
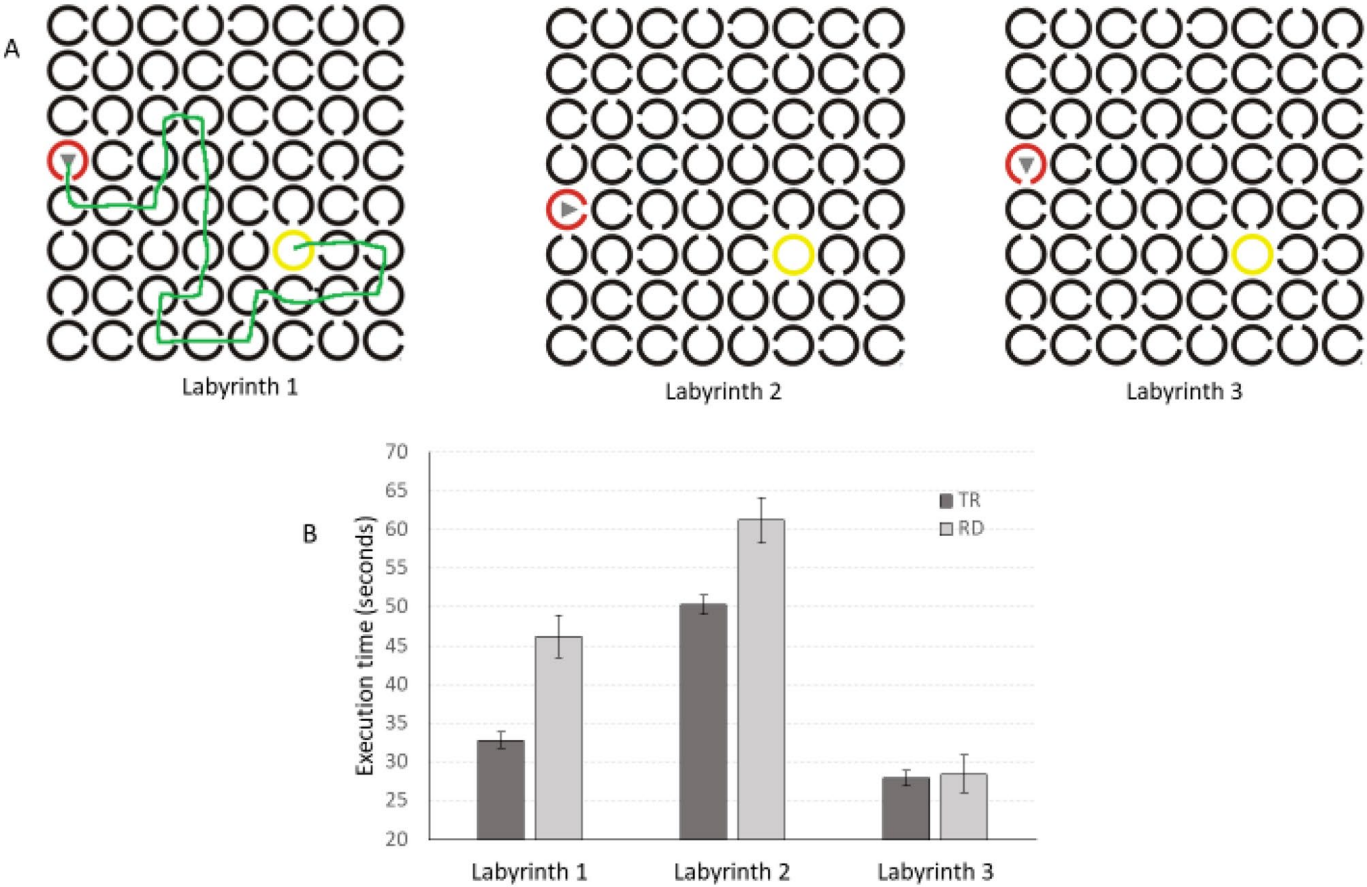
Panel (**A**) Representation of the three labyrinths that compose the visuo-spatial attention task. In the first labyrinth, an example of correct execution is represented; panel (**B**): Execution times (mean in seconds) in typical readers (TRs) and children with reading difficulties (RDs) in the three labyrinths. Error bars represent standard errors of the mean.

We supposed that a specific visuo-spatial attention deficit characterizes children with RD. The typical tasks used to index the pure visuo-spatial attention functioning in children with RD require complex behavioral^21^ and psychophysics procedures^20^ that are difficult to apply in a clinical setting. Thus, here we used a simple paper-and-pencil task composed of three Cs labyrinths (see Fig. 1, panel A). Efficient execution of the Cs labyrinths task requires multiple attentional mechanisms. Children must be able to rapidly zoom-in and zoom-out^19^ onto the different parts of the path, as well as disengage, move and engage their attentional focus^16,21–23^; see^58^ for a review. A large attentional focus could allow the child to analyze multiple items (Cs) of the labyrinth simultaneously. However, this efficient attentional procedure could be used only if noise^25^ or crowding of the peripheral stimuli^26^ is not excessive and if the visual attention span is adequate^31,59^. At the same time, a fast attentional zoom-in will allow children to rapidly disentangle the direction that must be serially selected item by item. Later, a rapid orientation of visual attention^21,36^ will allow quick analysis of subsequent path steps.

We hypothesized that the execution of this task allows the detection of differences between children with and without RD. We assumed that children with RD would have shown difficulty performing the attentional task, regardless of the different length of the first and second labyrinths.

To better understand the other cognitive mechanisms involved in this task, we tried to separate the role of visuo-attentional abilities from the possible effects of procedural learning and visuo-motor skills using three sheets in which the first and the third labyrinths path are equal. The visuo-spatial attention difficulties would have become less relevant if the path of the labyrinth had been the same as a labyrinth already solved. In this case, procedural learning would have played a more important role, than the visual-attentional abilities that would have been less engaged. Reducing the load on visuo-attentional skills by administering the first of the two labyrinths for a second time, we can control the effects linked not only to the procedural learning but also the visuo-motor coordination skills.

We tested this hypothesis in a large sample of primary school children (from the second to the fifth grade), administering word and pseudoword reading tasks and the three labyrinths composed by a series of Cs. We built the first and second labyrinths with different paths, whereas the first and third labyrinths were identical. Then, we measured the specific visuo-spatial attentional effect using the two labyrinths with different paths. Finally, our labyrinths task does not require a load of working memory processes, in contrast to the typical visual search tasks in which working memory of the target is highly involved.

## Material and methods

The entire investigation process was conducted according to the principles expressed in the Declaration of Helsinki. All participants provided written informed consent, and the Ethics Committee of the Department of General Psychology of the University of Padua approved all procedures.

### Participants

Three hundred ninety-eight children (188 males and 210 females; 8% left-handed) took part in our study. The children, partially evaluated in schools in different regions of Italy, attended from the second to the fifth grade of primary school (see Table 1). Children had normal or corrected to normal vision. No hearing difficulties or neurological deficits were reported. Clinicians evaluated children’s reading abilities using standardized word and pseudoword reading tasks (see the “Word and pseudoword reading tasks”). Based on their performance in standardized word and pseudoword reading tasks, they were classified as typical readers (TRs) or children with RD. A child was classified in the RDs group if they showed at least two performance measures (speed and/or accuracy) in word and/or pseudoword reading tasks -2 standard deviations (SDs) below the average scores calculated for a normative sample^60^. A subgroup of these children (n = 19) with RDs was recruited from clinical centers using the same reading disorders criteria. These children have already received a clinical diagnosis of developmental dyslexia based on the specific criteria established by the Italian Institution of health. These children may present other specific learning disabilities, but they did not receive other diagnoses of neurodevelopmental disorders. The other children were classified in the TRs group. A sample of 340 TRs (52% female) and 58 children with RDs (55% female) were tested (see Table 1).

**Table 1:** Sample size, chronological age (mean and standard deviation) and gender (number of males and females) characteristics of typical readers (TRs) and children with reading difficulties (RDs) in the different school grades.

	Typical readers (TRs)			Children with reading difficulties (RDs)			
School grades	Sample size	Age in years	Males/females	Sample size	Age in years	Males/females	t test
2	82	7.72 (0.31)	49/33	6	7.85 (0.40)	3/3	t_(86)_ = − 1.009 p = 0.316
3	85	8.75 (0.36)	39/46	21	8.75 (0.46)	10/11	t_(104)_ = 0.123 p = 0.903
4	87	9.56 (0.52)	39/48	18	9.7 (0.37)	7/11	t_(103)_ = 1.453 p = 0.149
5	86	10.65 (0.40)	35/51	13	10.75 (0.25)	6/7	t_(97)_ = − 1.015 p = 0.313
Total	340	9.34 (1.00)	162/178	58	9.23 (1.14)	26/32	t_(396)_ = − 0.851 p = 0.395

### Word and pseudoword reading tasks

Reading skills and phonological decoding abilities were measured using lists of standardized word and pseudoword reading tasks, respectively^60^. See Table 2 for the descriptive statistics of the reading tasks.

**Table 2:** Reading performance (means and standard deviations in Z score) in typical readers (TRs), children with reading difficulties (RDs) and reading performance differences between the two groups.

	Sample size	Word reading speed (Z score)	Word reading accuracy (Z score)	Pseudoword reading speed (Z score)	Pseudoword reading accuracy (Z score)
Typical readers (TRs)	340	0.018 (0.92)	0.06 (0.92)	− 0.10 (1)	0.02 (1.1)
Children with reading difficulties (RDs)	58	− 4.18 (3.42)	− 3.70 (3.54)	− 3.61 (4)	− 2.44 (1.75)
		t_(396)_ = 19.067 *p* < 0.001	t_(396)_ = 16.671 *p* < 0.001	t_(396)_ = 13.903 *p* < 0.001	t_(396)_ = 14.388 *p* < 0.001

### Cs labyrinth task

The task consists of three sheets of labyrinths. On each sheet, there is a square grid (8 × 8) of Cs (2.2 cm in diameter; the opening part of the C is about 0.5 cm), oriented in four cardinal directions. A red C with a triangle inside indicates the starting point, whereas a yellow circle indicates the endpoint. The participant was asked to draw a solid line from the triangle to the final circle using the opening of the black C to reach the adjacent C (see Fig. 1, panel A). In detail, the instructions given to the child were “In this test you must be able to drive this car (triangle) out of the labyrinths. The car can move from circle to circle, but it can only pass by the part where the circle is open, and then arrive in the consecutive circle, up to the yellow circle: you have to be fast and accurate”. Before the test, the child was shown an example sheet, where the administrator showed how to carry out the test, and a second sheet, where the participant carried out a training trial him-/herself. The three labyrinths were always administered in the same order to allow us to investigate the procedural learning skills. In particular, it is expected that the double administration of the same labyrinth path should cause an improvement in the second administration–an implicit facilitatory visuo-motor effect at the basis of procedural learning mechanism^50^.

Despite the path of the first labyrinth was composed of 21 passages and the path of the second labyrinth was composed of 36 passages, the attentional shifting index (measured as the ratio of total passages to the direction changes) was similar between them (first labyrinth: 12 direction changes with attentional shifting difficulty index = 1.75; second labyrinth: 21 direction changes with attentional shifting difficulty index = 1.71).

For each of the three labyrinths, the execution time in seconds and the number of errors were measured. It was not necessary for the pencil line to pass precisely inside the opening of the C. Each time the participant made a mistake entering in the wrong adjacent C, she/he restarted from the last correct circle.

Trained psychologists individually administered the reading and visuo-spatial attention tasks.

## Results

### Visuo-spatial attentional differences between TRs and children with RDs: analysis of (co)variance

Data analysis was performed using a 3 × 2 mixed analysis of variance (ANOVA) (3 labyrinths*2 groups: TRs and RDs children), where the three labyrinths are the repeated measures and the group is the between-subject factor. The dependent variable was the labyrinth execution times and errors (raw score). Considering that the participants were recruited from second to fifth grade of primary school, we decided to perform the same analysis, adding the school grade factor as a covariate.

#### Execution times

The first ANOVA on labyrinth execution times showed that the main effect of the labyrinth was significant: F_(2792)_ = 143.619, *p* < 0.001, partial η^2^ = 0.266. Post hoc (Bonferroni correction) revealed that the execution times of the three labyrinths were significantly different from each other (all ps < 0.001; execution time of labyrinth 1 mean = 34.77 s, SD = 21.02; labyrinth 2 mean = 51.88 SD = 22.91; labyrinth 3 mean = 29.99, SD = 18.92). Also, the group’s main effect was significant: F_(1396)_ = 13.402, *p* < 0.001, partial η^2^ = 0.033. The mean execution time of TRs (37 s, SD = 15.65) was shorter than that of children with RDs (45.29 s, SD = 17.41). In children with RD the execution time was 22% slower in comparison to TRs. The labyrinths x group interaction was also significant: F_(2792)_ = 8.684, *p* < 0.001, partial η^2^ = 0.021. To better understand this interaction, we run an ANOVA for each labyrinth. The main effects of group showed that the TRs group (first labyrinth mean = 32,82, SD = 19.98; second labyrinth mean 50.29, SD = 22.09; third labyrinth mean = 27.92, SD = 19.87) performed both first (F_(1395)_ = 21.056, *p* < 0.001, partial η^2^ = 0.05) and second (F_(1395)_ = 11.593, *p* = 0.001, partial η^2^ = 0.028) labyrinths significantly faster, compared to the RD group (first labyrinth mean = 46.19, SD = 23.36; second labyrinth mean = 61.23, SD = 25.5; third labyrinth mean = 28.45, SD = 12.06). Performance was not statistically different in the third labyrinth (F_(1395)_ = 0.039, *p* = 0.843, partial η^2^ < 0.001). Both TR (t_(339)_ = 3.318, *p* = 0.001 Cohen’s d = 0.180) and RD (t_(57)_ = 5.965, *p* < 0.001, Cohen’s d = 0.649) groups showed a significant improvement between the first and third labyrinth execution. Thus, children with RD were significantly slower only in the first and second labyrinth compared to TRs.

In the ANCOVA, with the same 3 × 2 (3 labyrinths*2 groups) analysis, in which we use the school grade as covariate, the main effect of school grade was significant: F_(1395)_ = 14.383, *p* < 0.001, partial η^2^ = 0.035. Also, the main effect of labyrinths was significant: F_(2790)_ = 34.301, *p* < 0.001 partial η^2^ = 0.080. The differences between the three labyrinths were still significant (all ps < 0.001). Labyrinth x school grade interaction was not significant: F_(2790)_ = 1.877, p = 0.154, partial η^2^ = 0.005. The main effect of groups was significant: F_(1395)_ = 15.085, *p* < 0.001, partial η^2^ = 0.037. Also, the labyrinths x group interaction was still significant: F_(2790)_ = 8.770, *p* < 0.001, partial η^2^ = 0.022. To better understand this interaction, we run an ANCOVA for each labyrinth. The main effects of group showed that the TRs group performed both first (F_(1395)_ = 22.084, *p* < 0.001, partial η^2^ = 0.053) and second (F_(1395)_ = 13.147, *p* = 0.001, partial η^2^ = 0.032) labyrinths faster compared to the RDs group, whereas the execution times in the third labyrinth did not differ (F_(1395)_ = 0.098, *p* = 0.754, partial η^2^ < 0.001; see Fig. 1). Thus, children with RD were significantly slower only in the first and second labyrinth compared to TRs independently of school grade.

#### Errors

A second (3 labyrinths*2 groups) mixed ANOVA, which considered the labyrinth error numbers as a dependent measure, was executed. The main effect of the labyrinth was significant: F_(2792)_ = 21.642, *p* < 0.001, partial η^2^ = 0.052. The number of errors was not different between the first (mean = 0.6, SD = 1.03) and second labyrinth (mean = 0.59, SD = 1, F_(1396)_ = 0.677, p = 0.411, partial η^2^ = 0.002), whereas the error numbers in both the first (F_(1396)_ = 31.4, *p* < 0.001, partial η^2^ = 0.073) and second labyrinth (F_(1,396)_ = 41.965, *p* < 0.001, partial η^2^ = 0.096) were significantly different from the third labyrinth (mean = 0.22, SD = 0.65). The main effect of the group was not significant: F_(1396)_ = 0.103, *p* = 0.748, partial η^2^ < 0.001. The group x labyrinth interaction was not significant: F_(2792)=_1.112, *p* = 0.329, partial η^2^ = 0.003.

The ANCOVA with the same design (3 labyrinths*2 groups), in which school grade was the covariate, showed that the main effect of school grade was not significant (F_(1.395)_ = 1.625, *p* = 0.203, partial η^2^ = 0.004). The school grade x labyrinth interaction was not significant (F_(2.790)_ = 0.197, *p* = 0.822, partial η^2^ < 0.001). The main effect of the labyrinth was significant (F_(2.790)_ = 4.692, *p* = 0.009, partial η^2^ = 0.012), whereas the main effect of the group (F_(1395)_ = 0.142, *p* = 0.707, partial η^2^ < 0.001), as well as the group x labyrinth interaction, were not significant (F_(2.790)_ = 1.122, *p* = 0.326, partial η^2^ = 0.003). Thus, children with RD were not significantly different to TRs when the errors in the labyrinth task were considered (see Table 3).

**Table 3:** Number of errors (mean and standard deviation) in the three labyrinths of children without (TR) and with reading difficulties (RD).

	Labyrinth 1 errors	Labyrinth 2 errors	Labyrinth 3 errors
TR group	0.57 (0.96)	0.59 (1.01)	0.23 (0.69)
RD group	0.72 (1.37)	0.60 (0.94)	0.16 (0.41)

Finally, these findings were not different when these analyses were performed considering only RDs selected in the schools excluding children with DD, showing that these results are not uniquely driven by the clinically diagnosed children with DD.

### Relationship between visuo-spatial attention and reading: partial correlation analysis

Besides investigating differences in visuo-spatial processing between children with and without RDs, we further investigated the correlation between individual visuo-spatial attentional abilities and reading skills, across our entire sample of children (n = 398).

**Figure 2:**
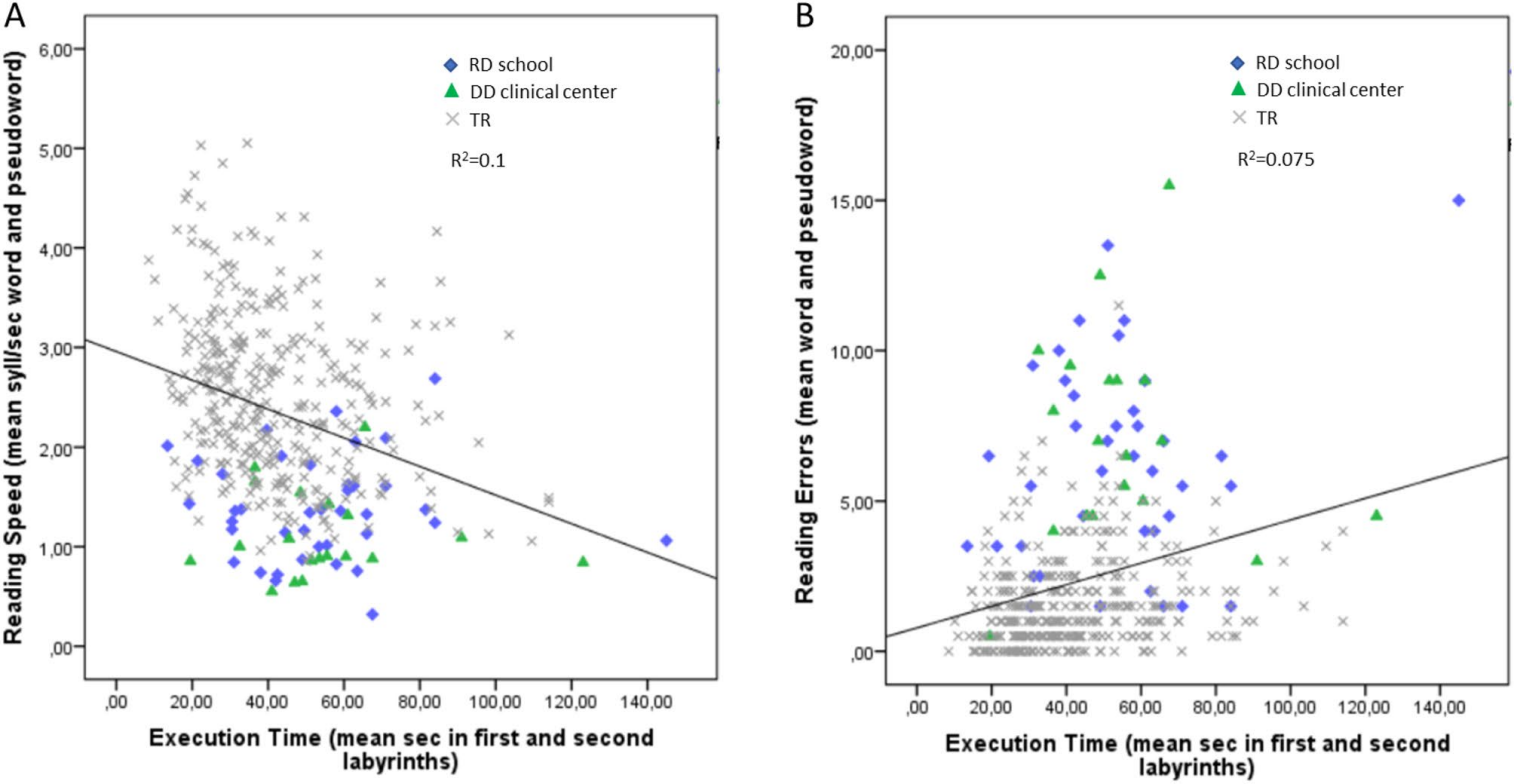
Panel (**A**) Representation of the bivariate correlation between the mean execution time in the first and second labyrinths and the mean of word and pseudoword reading speeds; Panel (**B**) Representation of the bivariate correlation between the mean execution time in the first and second labyrinths and the mean of word and pseudoword reading errors.

We carried out partial correlations between the execution time in the Cs labyrinths and reading speed (syllables for second) and errors, controlling for school grade. Significant correlations were found between the mean execution time in the first and second labyrinths and the mean of word and pseudoword reading speeds (r = − 0.28, *p* < 0.001; See Fig. 2 Panel A) and errors (r = 0.26, *p* < 0.001; See Fig. 2 Panel B). In contrast, no significant correlation was found between the execution time of the third labyrinth and the mean of word and pseudoword reading speeds (r = − 0.08, *p* = 0.113) and errors (r = − 0.03, *p* = 0.621).

A significant correlation was also found between the mean of errors in the first and second labyrinths with the mean of word and pseudoword reading errors (r = 0.19, *p* < 0.001), but not with speed (r = − 0.08, *p* = 0.113). No significant correlation was found between the number of errors in the third labyrinth and the mean of word and pseudoword reading speeds (*r* < 0.001, *p* = 0.994) and errors (r = − 0.016, *p* = 0.746).

In addition, to investigate the different cognitive mechanisms involved in the execution of the same visuo-motor paths, the correlation between the performance (errors and execution time) of the first and the third labyrinths was carried-out controlling for the school grade. Although the correlation between the mean of errors in the first and in the third labyrinth was significant (r = 0.12, *p* = 0.014), the same correlation was not significant (r = 0.08, *p* = 0.119) when the more sensitive execution times were analysed. These findings suggest that the same visuo-motor path executed the second time is able to measure a different cognitive mechanism, that is, procedural learning.

Moreover, the correlation between the pure visuo-spatial attention deficit controlling for procedural learning skills (measured through the delta of the execution time between 1st and 2nd administration of the same labyrinth) and reading speed (mean of syll/sec of word and pseudoword) was significant: (r = − 0.15, *p* = 0.003) also controlling for school grade. This result indicates that a more severe visuo-spatial attention deficit was linked with slower reading speed. Accordingly, this pure visuo-spatial attention deficit was also significantly correlated with the reading errors mean (r = 0.22, *p* < 0.001), indicating that a more severe attentional deficit was linked with more reading errors. The same visuo-spatial attention deficit calculated using the delta in errors between the 1st and 2nd administration was not significantly correlated with the reading speed (r = − 0.07, *p* = 0.14), whereas it was significantly correlated with the reading errors mean (r = 0.18, *p* < 0.001).

### Visuo-spatial attention deficit in children with RDs: individual data and odds ratio analysis

In the labyrinths 1 and 2, 34.5% (20/58) of children with RDs showed a visuo-spatial attention performance below one SD in comparison to the mean execution time of TRs (see Table 4 for normative date in TRs).

**Table 4:** Mean execution time (in seconds) and errors (mean and standard deviation) in labyrint one and two of the typical reader sample divided by school grade.

	2° grade (n = 82)	3° grade (n = 85)	4° grade (n = 87)	5° grade (n = 86)
Time (seconds)	44.11 (18.77)	44.64 (20.19)	37.42 (16.17)	38.3 (17.64)
Errors (number)	0.62 (0.76)	0.69 (0.87)	0.51 (0.82)	0.51 (0.80)

The odds ratio was 3.13 (95% confidence interval between 1.68 and 5.81), indicating a moderate connection between the presence of RDs and a pure visuo-spatial attention deficit.

## Discussion

A visuo-spatial attention dysfunction plays a pivotal role in the development of reading abilities hampering orthographic processing^16,17,26,28,29^; see^18^ for a meta-analysis). We found a significant difference between children with and without RDs in visuo-attentional skills using a simple paper-and-pencil task in which no auditory-phonological abilities were involved.

Computational models of reading assume a form of graphemic parsing to achieve the level of representation on which the grapheme-to-phoneme conversion mechanism operates. Visual input is segmented into single letters that are serially and individually processed^61^. Other models assume segmentation into sublexical units that are assigned to specific slots according to their position in the syllable^62–64^.

Regardless of how graphemic parsing is conceived, it requires primarily an efficient distributed visuo-spatial attention on entire letter-string, and then a focusing of visuo-spatial attention on each sublexical unit (single letter or letter cluster), inhibiting the flanking units^61^; see^18^ for a meta-analysis).

Distributed and focused visuo-spatial attention are implicated also in visual search tasks^65^. Indeed, it has been demonstrated that visual search abilities—without involving any phonological skills—are good predictors of future reading skills both in shallow and deep orthographies^26–29,34^, and visuo-spatial attention training by using action video games improves both visual search efficiency and reading skills in children with and without dyslexia^42,44^.

However, visual search tasks require not only distributed and focused visuo-spatial attention but also working memory for the visual target as well as a correct matching between the specific target and the focused candidate item (see^65,66^ for a review). Visual working memory is impaired in children with dyslexia^67^. Importantly, in the labyrinth task all elements (Cs) that compose the visual paths are sequential targets that should be processed without the involvement of working memory, minimizing the possible effect of visual working memory deficits in our visual task. Thus, our findings show that pure visuo-spatial attention difficulties—regardless of visual working memory skills—seem to characterize children with RD.

The possible causal relationship between visuo-spatial attention and reading acquisition has been critically discussed by Goswami^68^, because reading experience could directly affect visuo-spatial attention development. However, the absence of exclusive left-to-right attentional shifting characterising the reading direction in western orthographies, and the necessity of continue re-orienting of attention in all the directions in the Cs labyrinth task, excluded the possibility that this difference could be due to a simple practice effect linked to the habitual left-to-right attentional shifting trained during reading acquisition and consolidation^69^.

The selective difference in execution times between the two groups in the first and second labyrinth shows the importance of good visuo-spatial attention abilities, rather than a general speed of processing deficit. Indeed, in the first two labyrinths, participants were forced–for each C, or each chunk of Cs–to shift their visuo-spatial attention focus rapidly. Note that although the second labyrinth required a larger number of passages than the first (as indicated by slower execution times), the difference between the two groups was similar. It seems that the number of passages is not sensitive to the visuo-spatial attention impairment shown in children with RD. However, a more sensitive index of attentional shifting difficulty could be measured considering the ratio between the total number of passages and the number of passages that require a change in attentional shifting. The two labyrinths were similar in this attentional shifting difficulty index. These data could explain why the performance difference between the two groups was not different in labyrinths 1 and 2. To test this possible interpretation, we could increase the attentional shifting difficulty index in the Cs labyrinth task to improve the visuo-spatial attention sensitivity in the embedded visual condition^26,66^.

Independent from the group analysis, in which we chose a critical cutoff to divide children with and without RD, the results of the partial correlation analyses confirmed the relationship between visuo-spatial attentional processing and reading skills. Correlations between the execution times of the labyrinth task, word and pseudoword reading speeds and errors were significant only for the first two labyrinths that requested a higher level of visuo-spatial attentional abilities, confirming the specific link between fronto-parietal visuo-spatial attention and specialized occipito-temporal visual word form area^70,71^.

Finally, individual data analysis showed that about 40% of children with RD are impaired in visuo-spatial attentional mechanisms measured by labyrinths 1 and 2, indicating the presence of attentional dysfunction in children with RD. It should be noted that the sensitivity of our labyrinth task to visuo-spatial attentional disorder could be improved with regard to increasing lateral visual noise and stressing the specific orienting and zooming attentional mechanisms required during this simple paper-and-pencil task. Thus, the labyrinth task appears to be a good tool to detect the presence of visuo-spatial attentional deficits in primary school children with RD and in children with other neurodevelopmental disorders associated with RD^1^.

In the third labyrinth, performance was not different in the two groups. Although they were not directly informed that this labyrinth was identical to the first one, children with RD were able to improve their performance (i.e., take advantage of their previous experience), as seen in the TRs group. This result suggests that our sample of children with RD, in this task, shows adequate procedural learning skills and that the visuo-motor skills did not play a critical role in the determination of between-group differences found in the first two labyrinths. We cannot exclude that in other tasks, children with RD should show a procedural learning deficit^55^. It should be noted that difficulties in procedural learning that involve motor abilities seem to characterize children with language impairments rather than children with RD^72^. It could be speculated that developmental coordination and language disorders share impairments in procedural learning that could be associated with additional cerebellar or motor cortex deficits^50^. However, further studies are necessary to test this specific prediction.

Developmental coordination disorder and DD are often present in comorbidities^46,47^. Performance in the Labyrinth task, largely involving motor coordination abilities, could be influenced by the presence of a disorder in this area. Nevertheless, the difference between the first and third labyrinth could help clinicians to estimate the possible presence of a specific difficulty in visuo-spatial attention, motor skills or procedural learning. As shown in the correlation section, the visuo-spatial attention deficit indexes (measured through the delta of the execution time between 1st and 2nd administration of the same labyrinth) control not only the procedural learning skills, but also the visuo-motor skills intrinsically captured in the performance of the third labyrinth. A difficulty exclusively in the first two labyrinths can indicate a specific visual attentional difficulty. The presence of a deficit in the execution of the first two labyrinths combined with an absence of improvement between the execution time in the first and third labyrinth, could indicate a deficit in procedural learning or in motor coordination.

We could not consider the observed visuo-attentional deficit to be causally linked to the RD because of the cross-sectional design of this research^68^. However, a large overlap between the brain networks associated with the dynamic pattern of executing both reading and visuo-spatial attentional tasks has been observed^12,71,73^. Importantly, the structural connectivity networks associated with different aspects of skilled reading showed that interconnectivity between left hemisphere language and right hemisphere attentional areas underlies both lexical and sublexical reading^12^. Moreover, both training^26,33,34,36,37–41^; see^43^ for a recent review) and longitudinal^26,28,29,33,34,59^ studies have previously demonstrated the causal links between visuo-attentional deficits and RD.

Another limitation of this study is the absence of additional tasks that measured other possible neurocognitive deficits associated with RD in developmental dyslexia. Nevertheless, both training^33,36,37,74^ and longitudinal studies^26–28,33,34,59^ have previously demonstrated that the link between visuo-attentional disorders and RD was present also controlling for other neurocognitive (e.g., RAN and auditory-phonological) deficits typically associated with developmental dyslexia.

Since visuo-spatial attention is relevant for reading acquisition, and it is frequently dysfunctional in children with RD, its clinical evaluation is crucial for the correct identification of a specific training designed to improve RD in neurodevelopmental disorders. Considering that different visuo-spatial attention interventions can improve reading skills in children with RD^34,36,38,39,41,74–77^, see^43^ for a review), an efficient reading remediation program should integrate auditory-phonological and visuo-attentional interventions.

Finally, in the present study the possible role of IQ in reading acquisition was not considered. However, the results linking IQ and reading development in children with RD are inconsistent^78–80^.

In sum, we show that children with RD, compared to those with typical reading skills, appear to be characterized by a visual attentional deficit independent of visual working memory and motor procedural learning. In particular, visual spatial attentional deficits captured by our labyrinth task highlight that both distributed and focused spatial attention could be impaired in children with RD. These visual attentional mechanisms are fundamental for both lexical and sublexical reading pathways development^12,71,81,82^.

## Data Availability

Data will be share on request to corresponding Authors: sandrofranceschini@gmail.com; andreafacoetti@unipd.it.
